# Nightmares Fluctuate Across the Menstrual Cycle and May be More Pronounced in Women With Premenstrual Syndrome

**DOI:** 10.1002/brb3.70383

**Published:** 2025-02-28

**Authors:** Léa Dobler, Océane Richard, Emmanuelle Clerici, Emilie Stern, Michel Lejoyeux, Marie‐Pia d'Ortho, Julia Maruani, Pierre A. Geoffroy

**Affiliations:** ^1^ Département de psychiatrie et d'addictologie, AP‐HP, GHU Paris Nord, DMU Neurosciences Hôpital Bichat ‐ Claude Bernard Paris France; ^2^ Centre ChronoS GHU Paris ‐ Psychiatry & Neurosciences Paris France; ^3^ Child Psychiatry Department Necker‐Enfants‐Malades Hospital Paris France; ^4^ Université Paris Cité, Inserm, NeuroDiderot Paris France; ^5^ Service de Physiologie – Explorations Fonctionnelles et Centre du sommeil Hôpital Bichat, AP‐HP Paris France; ^6^ Institute for Cellular and Integrative Neurosciences CNRS UPR 3212 Strasbourg France

**Keywords:** dreams, nightmares, premenstrual dysphoric disorder, premenstrual syndrome, sleep wake disorders

## Abstract

**Introduction:**

Premenstrual syndrome (PMS) affects nearly half of women worldwide and is associated with sleep disturbances, though the specific relationship between PMS and nightmares remains underexplored. Clinical observations suggest a potential link, leading this study to investigate whether women with PMS experience more frequent or intense nightmares compared to those without PMS.

**Methods:**

We conducted a prospective case series of seven women experiencing nightmares, all of whom participated in weekly imagery rehearsal therapy (IRT) over 1‐month. Each participant completed the daily record of severity of problems (DRSP) to assess PMS symptoms and kept daily dream diaries throughout one menstrual cycle, tracking nightmare frequency, intensity, and emotional valence. The nightmare severity index (NSI) was administered at the beginning and end of the study. Descriptive analysis was used for the dream metrics, and the Wilcoxon signed‐rank test was employed to assess changes in NSI scores.

**Results:**

Women with PMS exhibited an increase in nightmare frequency during the premenstrual phase, but no formal statistical comparisons were made between PMS and non‐PMS groups regarding dream frequency or intensity. A significant reduction in NSI scores (*p* = 0.03) was observed across the entire sample, though this effect was not significant in the PMS subgroup.

**Conclusions:**

This case series is the first, to our knowledge, to provide detailed longitudinal data indicating that nightmare frequency may fluctuate across the menstrual cycle and could be more pronounced in women with PMS. While IRT effectively reduced nightmare severity overall, its specific impact on nightmares in women with PMS requires further research in larger studies.

## Graphical abstract

This graphical abstract illustrates the fluctuations in estradiol (dashed black line), progesterone (solid gray line), and nightmare frequency (red line) across the menstrual cycle. Estradiol peaks during the follicular phase, reaching its highest levels around ovulation (Day 14), while progesterone increases significantly during the luteal phase, following ovulation. Nightmare frequency shows a similar pattern, with a potential increase during the luteal phase, especially in the premenstrual period (the final days of the luteal phase prior to menstruation). The shaded area highlights the premenstrual phase, during which increased nightmare frequency has been observed in some cases. These findings suggest that variations in estradiol and progesterone levels may influence nightmare frequency, particularly in the premenstrual phase, though further research is needed to fully clarify this relationship.

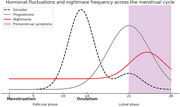

## 1 Introduction

Premenstrual syndrome (PMS) encompasses a range of clinically significant physical and psychological symptoms that occur during the luteal phase of the menstrual cycle, leading to substantial distress and functional impairment. PMS affects approximately 47.8% of women worldwide, with 20% experiencing symptoms severe enough to disrupt daily activities. Additionally, 80% to 90% of women exhibit at least one symptom of PMS (Gudipally and Sharma 2023; Direkvand‐Moghadam et al. 2014). The most severe form of PMS, premenstrual dysphoric disorder (PMDD), is recognized in the DSM‐5 and affects between 1.8% and 5.8% of menstruating women (Mishra et al. 2023; American Psychiatric Association. (2022). Common symptoms of PMS include fatigue, breast tenderness, headaches, weight gain, body aches, swelling of extremities, irritability, nervousness, mood swings, sadness, decreased concentration, hypersomnia or insomnia, and withdrawal from usual activities (Ryu and Kim 2015). Diagnostic criteria for PMS specify that symptoms occur up to 2 weeks before menstruation in most cycles, disappear shortly after the onset of menstruation, and are absent during most of the mid‐follicular phase (Halbreich et al. 2007). The pathophysiology of PMS is not fully understood, but is believed to involve hormonal fluctuations, particularly in estrogen and progesterone, and their interaction with neurotransmitters such as serotonin and gamma‐aminobutyric acid (GABA) (Yonkers et al. 2008; Epperson et al. 2002). These hormonal shifts may affect mood, cognition, and sleep patterns, potentially leading to the diverse symptoms observed in PMS.

Sleep disturbances are common among women with PMS and PMDD, particularly during the late luteal phase. Gender differences in sleep patterns typically emerge after puberty, likely influenced by hormonal changes. Women tend to have longer total sleep time and sleep latency, along with a reduced amount of slow‐wave sleep compared to men of the same age (Theorell‐Haglöw et al. 2018). Women with PMS/PMDD often perceive poor sleep quality during the late luteal phase, despite minimal disturbances in polysomnographic parameters (Baker and Lee 2018). They frequently report sleep‐related issues during the premenstrual period, including difficulty falling asleep, frequent nocturnal awakenings, nonrestorative sleep, and daytime consequences such as sleepiness, fatigue, concentration difficulties, decreased alertness, and impaired work performance (Jehan et al. 2016).

Nightmares are distressing dreams that typically occur during REM sleep, though they can also arise in other sleep stages, and are characterized by vivid, disturbing content that may lead to awakenings and significant distress. The literature mentions nightmares (Mauri 1990) or disturbing dreams (Baker and Lee 2018) occurring during the premenstrual period in patients with PMS, but no study has specifically examined the association between PMS and nightmares. Occasional nightmares are common in the general population, with a prevalence ranging from 35% to 45%, whereas nightmare disorder affects only 3% to 8% of the general population (Akkaoui et al. 2020). The etiology of nightmares involves genetic, neurobiological, and psychological factors. Neurobiologically, nightmares are linked to dysregulations in REM sleep, characterized by heightened brain activity and vivid dreaming, with the limbic system, particularly the amygdala, being highly active during REM sleep. Imbalances in neurotransmitters such as serotonin and norepinephrine can also exacerbate nightmare frequency and severity (Gieselmann et al. 2019). Psychologically, stress, trauma, and anxiety can trigger nightmares, reflecting unresolved emotional conflicts and maladaptive processing of distressing memories.

There is also a significant link between frequent nightmares or bad dreams and an increased risk of suicidal ideation and behavior. Nightmares can exacerbate feelings of distress and worsen depressive symptoms, which in turn can increase the risk of suicidal thoughts and actions (Pigeon et al. 2012). More generally, sleep complaints are associated with an increased risk of suicide independently of psychiatric disorders (Geoffroy et al. 2021). This association is particularly relevant in women with PMS or PMDD, as they are already at an increased risk for mood disturbances and suicidal behavior (Prasad et al. 2002). Recent literature also highlights how stressors, such as the COVID‐19 pandemic, can exacerbate PMS and other menstrual alterations, potentially contributing to sleep disturbances (Aolymat et al. 2023, 2022). These findings underscore the importance of considering comorbid psychological or psychiatric conditions when examining PMS‐related sleep disruptions, including nightmares. Given the high prevalence of PMS and its impact on sleep, understanding the relationships between PMS‐related nightmares and mental health outcomes, including suicidal crises, is crucial for developing effective interventions and improving the quality of life for affected women (Geoffroy et al. 2022).

Therefore, the primary objective of this first study was to investigate the relationship between PMS and the occurrence of nightmares. Using a sample of women who participated in an imagery rehearsal therapy (IRT) group for nightmare disorders and completed the daily record of severity of problems (DRSP) and daily dream diaries for over a month, we aim to address the gap in the current literature and provide clinically relevant insights into the interactions between PMS symptoms and sleep disturbances.

## 2 Methods

### 2.1 Population

Women experiencing nightmares, who were receiving care at the ChronoS Center of the GHU Paris—Psychiatrie et Neurosciences between August 2023 and April 2024, were examined in a clinical setting. This project entitled “Study of biomarkers of sleep and circadian rhythms in psychiatric disorders (Som‐Psy)” (principal investigator: PA Geoffroy) has been ethically approved (N°CER‐2020‐56) by the “Comité d'Evaluation de l'Ethique des projets de Recherche Biomédicale (CEERB) Paris Nord” (Institutional Review Board‐IRB 00006477 of the HUPNVS, Université Paris 7, AP‐HP). This investigation was designed as a prospective case series to explore the relationship between PMS and nightmares. Patients were given oral information, and a written note concerning the study protocol, and each received a no‐objection note.

Inclusion criteria were as follows: women aged 18 to 45, proficient in written and spoken French, and having regular menstrual cycles (18 to 32 days). Clinical and sociodemographic information was collected using standardized clinical interviews, including gynecological and obstetric history and current contraception methods.

Exclusion criteria included pregnancy, perimenopause or menopause, and irregular menstrual cycles (e.g., < 21 or > 35 days).

### 2.2 Assessments

To assess premenstrual symptoms, the DRSP scale was used (Endicott et al. 2005). This prospective scale includes 24 items (21 items grouped into 11 distinct symptoms and 3 items related to functional impact). Each item is rated daily on a severity scale from 1 (not at all) to 6 (extreme). The main advantage of this prospective scale is that it aligns with DSM‐5 diagnostic criteria for PMDD, providing information on symptom severity and functional impairment across menstrual cycle phases. It is widely recommended in the literature for evaluating premenstrual symptoms (Hall and Steiner 2015; O'Brien et al. 2011). The DRSP was translated into French by Léa Dobler, with an additional translation and back‐translation performed by Emmanuelle Clerici, Emilie Stern, and Pierre Alexis Geoffroy. The Carolina premenstrual assessment scoring system (C‐PASS) algorithm, used for diagnosing PMDD and the subthreshold research diagnosis of menstrually‐related mood disorder (MRMD), was employed when feasible. This method requires daily evaluation over two menstrual cycles using the DRSP scale (Eisenlohr‐Moul et al. 2017).

The frequency and characteristics of nightmares were evaluated using a dream diary, which was completed daily by the patients to better characterize their dream content, including emotional intensity and valence. Additionally, the nightmare severity index (NSI), which has a scoring range from 0 to 20, was also completed by patients multiple times during the observation period. The NSI assesses various aspects of nightmares, including frequency, emotional impact, and effects on daytime functioning, with higher scores indicating greater severity. The NSI is freely accessible and can be downloaded from the original publication by Geoffroy et al. (Geoffroy et al. 2024).

### 2.3 Statistics

Descriptive statistics, including means, medians, and standard deviations, were used to summarize the demographic and clinical characteristics of the sample, as well as the dream‐related variables such as the frequency of nightmares and dreams, intensity, and emotional valence. The premenstrual period was defined as the 7 days preceding menstruation, including the first day of menstruation. Comparisons were made between the premenstrual period and the full menstrual cycle, which was defined as the interval from 7 days before menstruation (Day 7) through the 31st day of the cycle (Day 31).

Nightmare and dream frequencies were calculated as the proportion of days with nightmares or dreams relative to the total number of days with available data. This approach accounted for any missing entries in the dream diaries. Missing data were not imputed, and it was assumed that no nightmares or dreams occurred on days with incomplete records.

The primary analysis focused on descriptive measures, examining the variations in dream and nightmare frequencies, intensity, and emotional valence between the premenstrual period and the entire menstrual cycle. Inferential statistics were limited to the comparison of the NSI scores between the 1st and 4th weeks of treatment, where the Wilcoxon signed‐rank test was employed to assess changes over time. This analysis was performed for the entire sample as well as for the subgroup of patients who met criteria for PMS, using R (version 4.4.1).

No additional hypothesis testing was conducted to compare patients with and without PMS given the small sample size and exploratory nature of the study.

## 3 Results

Seven patients were selected for inclusion in the study based on their age and menstrual cycle criteria, as well as their participation in the ChronoS Center's weekly nightmare therapy group for approximately 1‐month. The demographic and clinical characteristics of the seven patients are summarized in Table 1. The mean age of the participants was 31 years (SD = 7.5 years). All patients experienced nightmares, as they were participating in an IRT group at the ChronoS Center. Although not all patients had a formal diagnosis of nightmare disorder according to the ICSD‐3 (International Classification of Sleep Disorders, Third Edition) criteria (American Academy of Sleep Medicine 2024), their recurrent dysphoric dreams and significant daily functioning impairment, which led them to seek treatment, strongly suggest that they met clinical thresholds for the disorder. Additionally, some patients had comorbid conditions, such as major depressive disorder, which may complicate the diagnostic picture.

**TABLE 1:** Summary of subject demographics and medical history.

Subject	Age (years)	Menstrual status	Gravidity and Parity (G&P)	Medication treatments (including contraception)	Medical history (including psychiatric history et sleep disorders)
**1** **(PMS)**	41	Regular menses by history	G2P0 (2 spontaneous miscarriages)	Dietary supplement (TOKO 500 mg, OVUNOL OMEGA 3)	Chronic insomnia disorder Nightmare disorder
2	31	Regular menses by history	G0P0	Venlafaxine 150 mg daily	Major Depressive Episode (MDE): under treatment, evolving for several months Nightmares
3	30	Regular menses by history	G0P0	Oral contraceptive (YAZ, éthinylestradiol 0.02 mg, drospirénone 3 mg)	History of major depressive episode Nightmares
**4** **(PMS)**	22	Regular menses by history	G0P0	Venlafaxine 75 mg daily	Generalized anxiety disorder Major depressive episode in remission Nightmare disorder
5	28	Regular menses by history	G0P0	Fluoxetine 60 mg daily Melatonin 1 mg at bedtime Folic acid 5 mg daily Oral contraceptive (MISOLFA, éthinylestradiol 0.03 mg, diénogest 0.2 mg)	Major depressive episode in partial remission Nightmare disorder Endometriosis diagnosed since 2015
6	41	Regular menses by history	G0P0	Iron supplementation	Anxiety disorder Nightmares Hashimoto's thyroiditis (supplemented for 1‐year, discontinued several years ago)
7	24	Regular menses by history	G0P0	Sertraline 200 mg daily Abilify 15 mg daily Melatonin LP 2 mg at bedtime Largactil 25–50 mg per day as needed	Post‐traumatic stress disorder Nightmares

Abbreviation: PMS, premenstrual syndrome.

The available data, based on the scales completed by the patients, allowed for the analysis of one menstrual cycle per patient. Among the seven patients, two met the criteria for PMS according to the C‐PASS algorithm, five did not, and one patient had insufficient data to apply the C‐PASS algorithm. Notably, patient 4 had previously reported a history of PMS in her medical records, which aligned with the findings during the studied cycle. The median cycle length was 29 days (range: 27–29 days); however, the precise cycle length could not be determined for three patients due to missing data, and for one patient, this measure was not applicable due to continuous contraceptive pill use.

The number of days with available data varied among patients. For the premenstrual period (Days 7 to 1), data were available for 4 to 8 days depending on the patient, while for the full menstrual cycle (Days 7 to 31), the available data ranged from 13 to 38 days. Frequencies of dreams and nightmares were calculated based on the available data for each patient, and missing days were not included in the analysis.

Six of the seven patients showed a variation in their dream content across the premenstrual period and the entire menstrual cycle, with differences in the frequency of dreams and nightmares. Four patients (1, 3, 4, and 5) experienced more nightmares during the premenstrual period than over the full cycle, while two patients (2 and 7) showed no change in nightmare frequency but had more frequent dreams during the premenstrual period.

**FIGURE 1:**
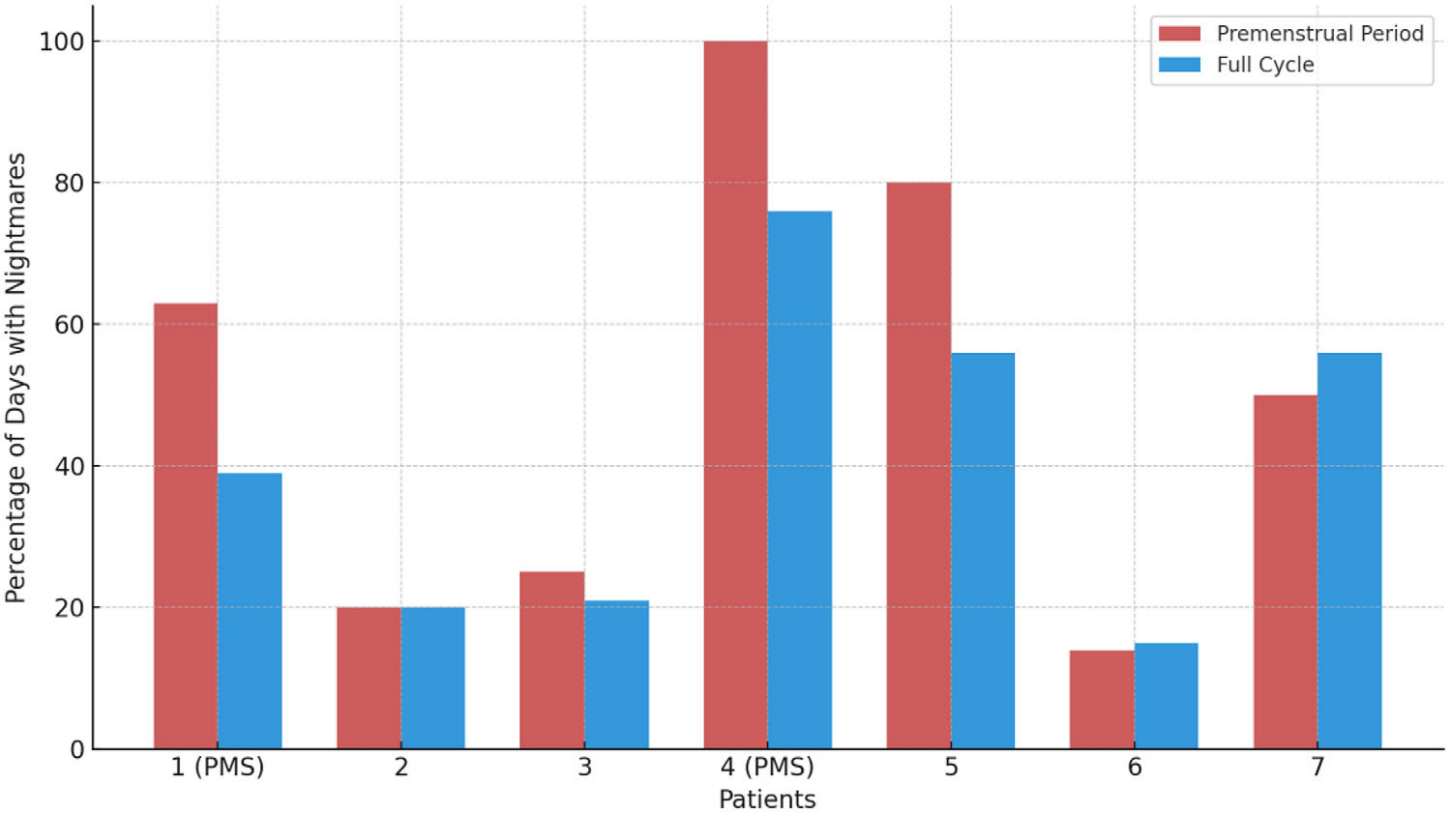
Nightmare frequency during the premenstrual period and full menstrual cycle (*n* = 7). Percentage of days with nightmares for each patient during the premenstrual period (red) and the full menstrual cycle (blue). Patients diagnosed with premenstrual syndrome (PMS) are indicated.

Patient 4, who met PMS criteria, experienced nightmares on all premenstrual days, though her overall dream frequency remained similar throughout the cycle. Patient 1, also in the PMS group, had more nightmares during the premenstrual period (63% of days vs. 39% over the full cycle). However, she reported fewer dreams during the premenstrual period (75% vs. 84% for the full cycle). Patient 5 reported a higher frequency of nightmares in the premenstrual period (80% vs. 56%), but her dream frequency remained identical throughout the cycle. Patient 3 reported fewer dreams overall during the premenstrual period but experienced a slightly higher proportion of nightmares during this phase. Figure 1 illustrates the differences in nightmare frequency across the premenstrual period and full menstrual cycle for all patients, highlighting a noticeable increase during the premenstrual phase, particularly in patients with PMS.

The intensity and emotional valence of dreams varied between patients. While most patients reported similar emotional valence between the premenstrual period and the full cycle, patient 3 reported more negative dream valence during the premenstrual period (1/10 compared to 3.8/10). Additionally, patient 4, who was diagnosed with PMS, noted increased dream intensity during the premenstrual period compared to the full cycle (6.8/10 vs. 5.7/10). Table 2 provides a comprehensive summary of the data.

**TABLE 2:** Summary of dream activity metrics during the premenstrual period and full menstrual cycle (*n* = 7).

Subject	Days with nightmares (premenstrual/month, %)	Days with dreams (premenstrual/month, %)	Average dream intensity (0–10) (premenstrual/Month)	Average dream valence (0–10) (premenstrual/month)
**1** **(PMS)**	5/15 (63%/39%)	6/32 (75%/84%)	4.8/5.1	4.3/4.3
2	1/5 (20%/20%)	5/17 (100%/68%)	5.4/6	3.4/3.2
3	2/7 (25%/21%)	0/11 (0%/32%)	9/6.2	1/3.8
**4** **(PMS)**	8/19 (100%/76%)	2/6 (25%/24%)	6.8/5.7	3.3/3.3
5	4/18 (80%/56%)	5/32 (100%/100%)	7.4/6.8	2.9/2.9
6	1/2 (14%/15%)	7/13 (100%/100%)	4.7/5.9	3.2/4.4
7	2/10 (50%/56%)	2/7 (50%/39%)	8/7.6	9.3/8

*Note*: Premenstrual period: Day 7 to the first day of menstruation. Full menstrual cycle: Day 7 to Day 31, valence scale: 0 = very negative, 10 = very positive. Percentages represent the number of days relative to the total number of days with available data for each period.

Abbreviation: PMS, premenstrual syndrome.

**FIGURE 2:**
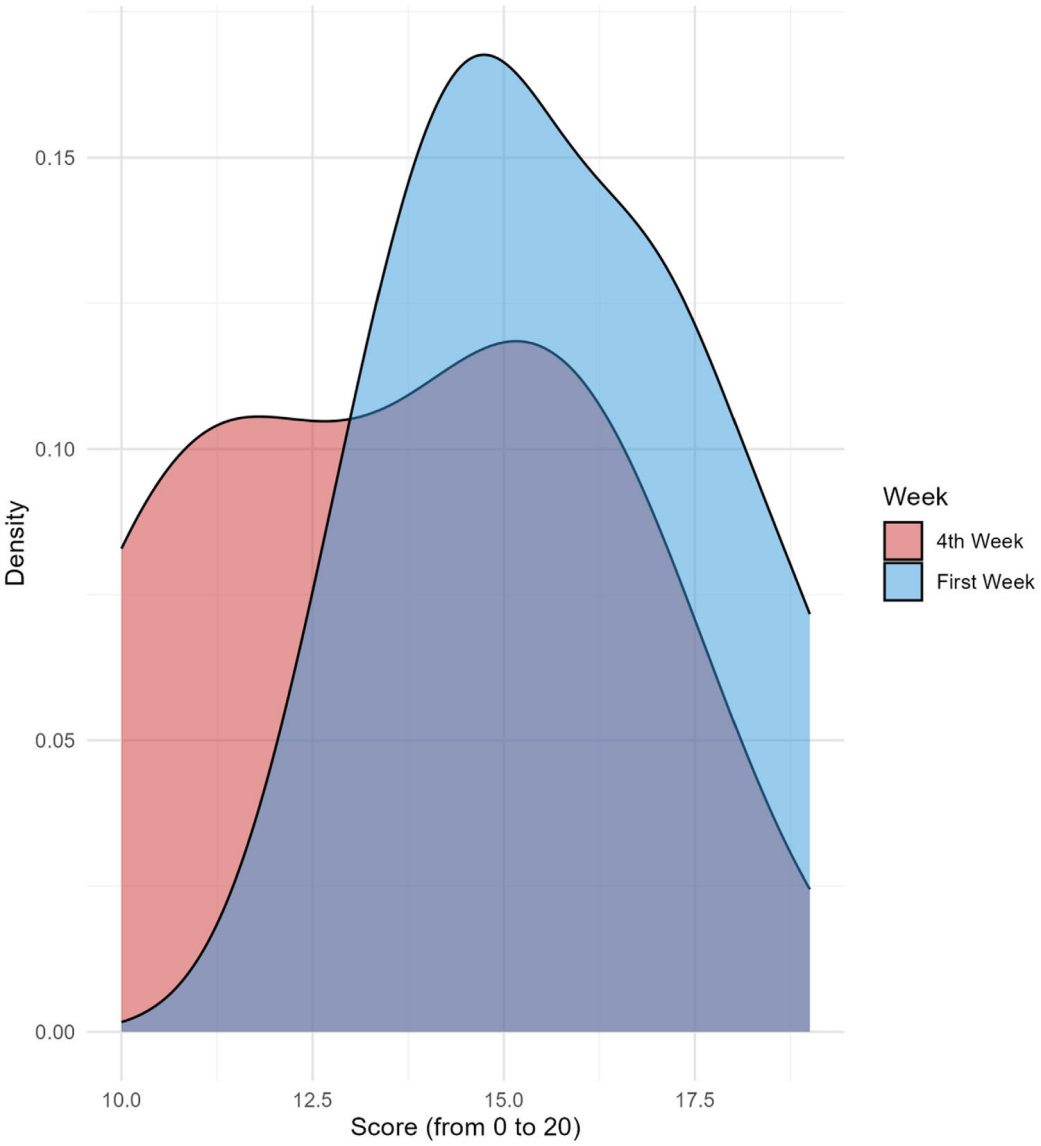
Distribution of nightmare severity index (NSI) scores during the 1st and 4th weeks. Density plot of NSI scores recorded during the 1st (blue) and 4th (red) weeks of the menstrual cycle. The *x*‐axis represents NSI scores (ranging from 0 to 20), while the *y*‐axis indicates density.

Finally, the NSI scores were assessed at both the 1st and 4th weeks of treatment. The NSI showed a statistically significant reduction across the entire sample, with a Wilcoxon *p* value of 0.03142, indicating a significant decrease in nightmare severity over time. The mean NSI score dropped from 15.71 (SD = 2.06, range = 12–19) in Week 1 to 13.57 (SD = 2.64, range = 10–17) in Week 4. The density plot (Figure 2) illustrates this shift, with the peak for Week 1 centered around 15. In Week 4, a more complex distribution emerged, featuring two peaks: one slightly above 15 and another around 12.5. This pattern highlights an overall improvement, though the score distribution in Week 4 is broader compared to Week 1. However, when focusing on the two patients with PMS, the comparison between NSI scores at Week 1 and 4 yielded a *p* value of 0.5, suggesting no significant change in nightmare severity for these patients.

## 4 Discussion

Although previous research had noted that women with severe PMS often report more disturbing dreams during the premenstrual week (Nowakowski et al. 2013; Meers and Nowakowski 2020), this is the first case series, to our knowledge, that provides detailed longitudinal data documenting both the frequency and intensity of nightmares across the menstrual cycle in women with PMS. Qualitative analysis revealed fluctuations in dream content, including nightmare frequency and intensity, between the premenstrual period and the entire menstrual cycle. Both patients with PMS experienced an increased nightmare frequency during the premenstrual phase. In particular, patient 4 reported nightmares on all premenstrual days and an increase in dream intensity during this phase. Moreover, emerging evidence suggests that stress‐related menstrual alterations can exacerbate sleep disturbances. Polese et al. found that students with new‐onset menstrual alterations and PMS during the pandemic experienced increased insomnia symptoms and nightmares (Polese et al. 2024). This highlights the interconnectedness of mood, stress, and menstrual function in disturbed dreaming. Although our cohort was smaller, our observations similarly suggest that PMS may heighten vulnerability to nightmares, particularly under heightened stress. Future research with larger cohorts is necessary to confirm these preliminary findings.

Recruiting patients for this study proved to be particularly challenging. The inclusion criteria required women within a specific age range, who were experiencing nightmares severe enough to warrant treatment, and who were willing to complete detailed daily assessments over a full menstrual cycle. These constraints, along with participation in a therapy group, limited the sample size, making it difficult to assemble a larger cohort.

Research has shown that biological sex and sex steroids significantly impact sleep patterns and disturbances, particularly during the luteal phase of the menstrual cycle when hormone levels fluctuate (Mong and Cusmano 2016). The influence of sex hormones like estrogen and progesterone on sleep architecture underscores the importance of considering sex‐specific factors in studies of sleep disturbances. In this study, only two of the seven patients were using a combined estrogen‐progestin contraceptive pill, neither of whom had PMS. Given that hormonal contraceptives can influence PMS symptoms and potentially modulate sleep disturbances associated with PMS, this factor could have contributed to the variability in sleep experiences observed among the patients. Although a recent meta‐analysis suggests that hormonal contraceptive use does not have a consistent effect on sleep (Bezerra et al. 2023), individual variability and specific formulations may still play a role in how sleep disturbances manifest in women with PMS, indicating a need for further research to explore these interactions.

The significant reduction in the NSI across the entire sample suggests that the intervention, likely IRT, was effective in decreasing nightmare severity. IRT is a well‐established treatment for nightmares, recommended by the American Academy of Sleep Medicine for nightmares associated with post‐traumatic stress disorder (PTSD) as well as idiopathic nightmares (Morgenthaler et al. 2018). However, the lack of significant improvement in NSI scores for the two patients with PMS suggests that this subgroup may respond differently to the intervention. Although the sample size was small, the lack of change in NSI scores for patients with PMS indicates that further research is needed to determine if specific therapeutic adjustments are required for PMS patients.

An additional consideration is the use of antidepressants in the study population. selective serotonin reuptake inhibitors (SSRIs) are commonly recommended for the treatment of PMS symptoms, including mood swings and irritability, as highlighted in the Cochrane review on this topic (Brown et al. 2009). In this study, two patients were taking SSRIs, and two others were on a serotonin‐norepinephrine reuptake inhibitor (SNRI), meaning that more than half of the sample was on antidepressant treatment. Notably, venlafaxine, the SNRI used by two patients in this study (including one with PMS), has been associated with vivid dreams (Doghramji and Jangro 2016), potentially influencing nightmare frequency and confounding the observed changes. Future larger studies should systematically control for PMS‐directed medications to clarify their impact on nightmare expression.

The differential response observed in the PMS subgroup might be attributed to the complex interactions between hormonal fluctuations, antidepressant use, and sleep disturbances, which are often exacerbated during the premenstrual phase. These fluctuations could influence the effectiveness of interventions like IRT, highlighting the need for personalized treatment approaches for patients with PMS. Future studies should examine larger cohorts and consider alternative or supplementary therapeutic strategies, taking into account the potential effects of concurrent antidepressant therapy.

The study's limitations, particularly the small sample size and the analysis of only a single menstrual cycle per patient, restrict the generalizability of the findings. Larger studies, ideally including multiple menstrual cycles per patient, would provide a more comprehensive understanding of the relationship between PMS and nightmares.

Notably, nightmares are closely tied to anxiety traits and emotional processing (Sikka et al. 2018; Gratton et al. 2024), and they share genetic risk factors with psychiatric and sleep‐related traits (Ollila et al. 2024). Additionally, they have been widely studied in the context of PTSD psychodynamic perspectives (Polese and Fagioli 2024). Our exploratory findings further suggest that PMS may be another factor that heightens vulnerability to nightmares; however, replication in larger samples is needed.

Nonetheless, this is the first case series, to our knowledge, that systematically documents both the frequency and intensity of nightmares across the menstrual cycle, highlighting the need for larger studies to confirm these menstrual fluctuations in nightmare occurrence. Additionally, investigating whether women with PMS require tailored interventions, especially considering the effects of concurrent antidepressant use on sleep and dreaming, would be essential in future research.

These findings have broader clinical implications for women suffering from both PMS and nightmares. Understanding the interactions between menstrual cycle phases, sleep disturbances, and mental health is crucial for developing effective, personalized treatment strategies. Clinicians should consider the potential impact of PMS on sleep and incorporate integrated approaches that address both PMS symptoms and nightmares. Recent intervention studies have shown that computer‐based stress inoculation training (SIT) can effectively target mental health factors (e.g., anxiety, depression) in individuals with PMS or dysmenorrhea (Zolfaghary et al. 2024; Dailer et al. 2024). Implementing similar approaches for women experiencing both PMS and frequent nightmares could be beneficial, as improving underlying mental health may help reduce nightmare occurrence and severity. Further research is warranted to explore tailored therapeutic strategies that address this overlap.

Moreover, it is essential to recognize that women are often underrepresented in clinical research, leading to gaps in knowledge and disparities in treatment outcomes. This underrepresentation extends to various aspects of women's health, including those uniquely affected by the menstrual cycle. As a result, critical factors like the menstrual cycle, which significantly influence women's health, are frequently overlooked in studies. This lack of consideration contributes to a scarcity of tailored treatment strategies for conditions such as PMS. Research highlights that women's health issues, including but not limited to sleep disturbances, are less studied and less understood, contributing to gender disparities in healthcare (Holdcroft 2007; Garcia‐Sifuentes and Maney 2021). Addressing these gaps through more inclusive research that accounts for the unique physiological aspects of women, such as the menstrual cycle, is essential for improving healthcare outcomes.

## 5 Conclusion

This case series is the first, to our knowledge, to provide detailed evidence that nightmare frequency may fluctuate across the menstrual cycle and could be more pronounced in women with PMS. While IRT showed overall effectiveness in reducing nightmare severity across the sample, patients with PMS did not demonstrate significant improvement, suggesting that PMS may require tailored therapeutic interventions. Given the small sample size and exploratory design, future research with larger cohorts is essential to validate these findings and further investigate the specific interactions between PMS, nightmare characteristics, and treatment responses.

## Author Contributions

**Léa Dobler**: conceptualization, investigation, writing–original draft, methodology, data curation, formal analysis. **Océane Richard**: formal analysis, visualization, writing–review and editing. **Emmanuelle Clerici**: investigation, writing–review and editing, methodology. **Emilie Stern**: writing–review and editing, methodology, investigation. **Michel Lejoyeux**: writing–review and editing. **Marie‐Pia d'Ortho**: writing–review and editing. **Julia Maruani**: writing–review and editing, methodology, investigation. **Pierre A Geoffroy**: writing–review and editing, conceptualization, supervision, project administration, validation.

## Conflicts of Interest

The authors declare no conflicts of interest.

### Peer Review

The peer review history for this article is available at https://publons.com/publon/10.1002/brb3.70383.

## Data Availability

The data that support the findings of this study are available from the corresponding author upon reasonable request. The data are not publicly available due to privacy or ethical restrictions.
